# Oligosaccharyltransferase: A Gatekeeper of Health and Tumor Progression

**DOI:** 10.3390/ijms20236074

**Published:** 2019-12-02

**Authors:** Yoichiro Harada, Yuki Ohkawa, Yasuhiko Kizuka, Naoyuki Taniguchi

**Affiliations:** 1 Department of Glyco-Oncology and Medical Biochemistry, Osaka International Cancer Institute, 3-1-69 Otemae, Chuo-ku, Osaka 541-8567, Japan; yoharada3@mc.pref.osaka.jp (Y.H.); yuki34@mc.pref.osaka.jp (Y.O.); 2 Center for Highly Advanced Integration of Nano and Life Sciences (G-CHAIN), Gifu University, Gifu 501-1193, Japan; kizuka@gifu-u.ac.jp

**Keywords:** endoplasmic reticulum, N-glycosylation, oligosaccharyltransfease, tumors

## Abstract

Oligosaccharyltransferase (OST) is a multi-span membrane protein complex that catalyzes the addition of glycans to selected Asn residues within nascent polypeptides in the lumen of the endoplasmic reticulum. This process, termed N-glycosylation, is a fundamental post-translational protein modification that is involved in the quality control, trafficking of proteins, signal transduction, and cell-to-cell communication. Given these crucial roles, N-glycosylation is essential for homeostasis at the systemic and cellular levels, and a deficiency in genes that encode for OST subunits often results in the development of complex genetic disorders. A growing body of evidence has also demonstrated that the expression of OST subunits is cell context-dependent and is frequently altered in malignant cells, thus contributing to tumor cell survival and proliferation. Importantly, a recently developed inhibitor of OST has revealed this enzyme as a potential target for the treatment of incurable drug-resistant tumors. This review summarizes our current knowledge regarding the functions of OST in the light of health and tumor progression, and discusses perspectives on the clinical relevance of inhibiting OST as a tumor treatment.

## 1. Introduction

**Figure 1:**
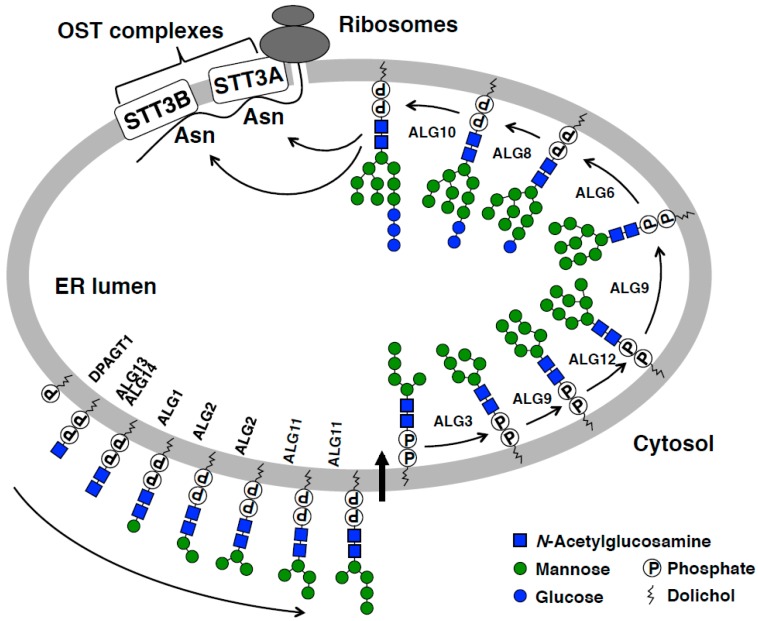
A model of N-glycosylation in the endoplasmic reticulum (ER). The biosynthesis of dolichol-linked oligosaccharides (DLOs) is initiated on the cytosolic side of the ER membrane by a series of membrane-anchored glycosyltransferases. A DLO intermediate (Man_5_GlcNAc_2_-PP-dolichol) is then transported to the ER lumen and further modified with mannose (Man) and glucose (Glc) units, resulting in the synthesis of the fully assembled DLO (Glc_3_Man_9_GlcNAc_2_-PP-dolichol). OST transfers the glycan moiety of DLO en bloc to amide group of the side chain of Asn residue within consensus sequences (Asn-Xaa-Ser/Thr, Xaa ≠ Pro), thus forming an N-glycosidic linkage. Mammalian cells that express STT3A and STT3B as catalytic subunits of OST and incorporated into distinct OST complexes (STT3A-OST and STT3B-OST). N-glycosylation reaction catalyzed by STT3A-OST is coupled with translation and translocation of nascent polypeptides (co-translational N-glycosylation), whereas STT3B-OST mediates the N-glycosylation of sites that are skipped by STT3A-OST (post-translational N-glycosylation) [17].

Most, if not all, of the secretory and membrane proteins synthesized in the endoplasmic reticulum (ER) of eukaryotes are modified with N-glycans (Figure 1) [1]. This post-translational protein modification is highly conserved throughout evolution and is catalyzed by the oligosaccharyltransferase (OST) complex [2]. The biological relevance of N-glycosylation has been elucidated in a number of genetic and biochemical studies that demonstrate that N-glycosylation plays an indispensable role in protein folding, degradation, trafficking, cell signaling, and intercellular communication [3,4,5].

When glycoproteins exit the ER and traffic through the Golgi apparatus for maturation, the N-glycans are subjected to dramatic structural remodeling by the concerted action of various glycosyltransferases and glycosidases [5]. The expression and function of these enzymes are tightly regulated by genetic and epigenetic factors, as well as by the supply of acceptor and donor substrates, which are often dysregulated in tumor cells [6,7]. As a consequence, tumor cells express aberrant N-glycans, which have been shown to be associated with malignancy and poor prognosis. The correlation between tumor progression and the terminal modification of N-glycans has long been an extensive focus of basic and clinical research studies in glycobiology and have been sophisticatedly reviewed elsewhere [6,7,8,9,10,11,12,13,14,15,16]. In contrast, the association of OST with tumor progression has only recently been explored. In this review, we summarize the functions of OST in health and diseases with a focus on genetic disorders and tumors.

## 2. Overview of *N*-Glycosylation in the ER

N-glycosylation in the ER is divided into glycan biosynthesis and transfer phases (Figure 1) [3,18,19]. Dolichol-linked oligosaccharides (DLOs) are glycolipids that function as glycan donor substrates for N-glycosylation. The biosynthesis of DLOs begins on the cytosolic side of the ER membrane and ends in the luminal side. In this process, monosaccharides are assembled onto dolichyl phosphate one at a time directly or indirectly from nucleotide sugars. The fully assembled DLO consists of three glucose (Glc), nine mannose (Man), and two *N*-acetylglucosamine (GlcNAc) residues that are covalently linked to dolichyl pyrophosphate. Once assembled, the tetradecaoligosaccharide (Glc_3_Man_9_GlcNAc_2_) of DLO is transferred by OST en bloc to Asn residues within the consensus sequences (Asn-Xaa-Ser/Thr, Xaa ≠ Pro) of nascent polypeptides. Genes that are involved in N-glycan biosynthesis in the ER are highly conserved in eukaryotes, whereas eubacteria and archaebacteria use different sets of genes to synthesize N-glycans [20,21,22]. It is important to note that the catalytic subunit of OST, that is, STT3, is evolutionarily conserved among the three domains of life [2]. In mammals, the *STT3* gene is duplicated and the gene products (STT3A and STT3B) are expressed to mediate N-glycosylation in a mutually complementary manner (see below) (Figure 1) [23].

## 3. OST and Its Action

**Figure 2:**
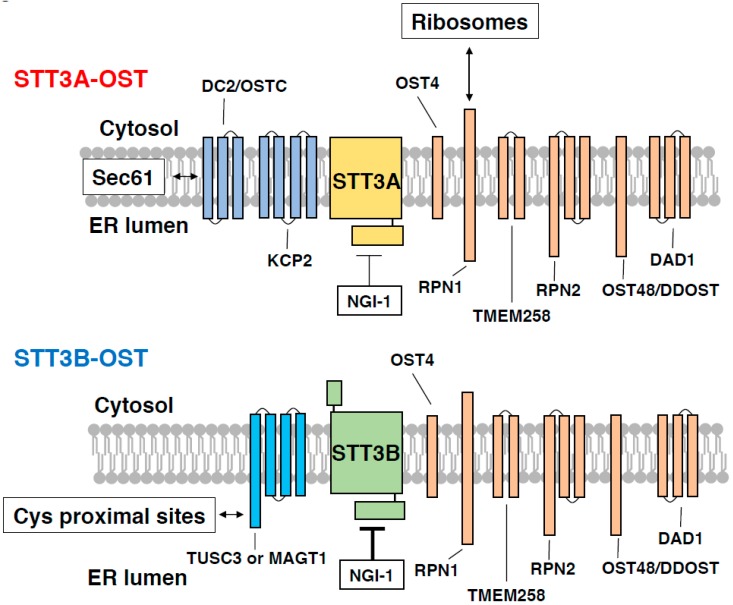
Subunit composition of STT3A-OST and STT3B-OST. STT3A-OST (upper side) and STT3B-OST (lower side) contain six shared subunits (RPN1, RPN2, DAD1, OST48, OST4, and TMEM258; shown in orange) and specific subunits (DC2/OSTC and KCP2 for STT3A-OST; shown in dark blue, and TUSC3 and MAGT1 for STT3B-OST; shown in cyan). The cytosolic domain of RPN1 in complex with STT3A-OST makes contact with the 60S subunit of membrane-bound ribosomes [35]. In contrast, DC2/OSTC mediates interaction between STT3A-OST and Sec61 protein-conducting channel [35], allowing co-translational N-glycosylation. STT3B-OST contains either one of TUSC3 or MAGT1, which has an oxidoreductase activity and facilitates N-glycosylation of Cys proximal sites [33]. N-glycosylation inhibitor 1 (NGI-1) inhibits STT3B-OST more efficiently than STT3A-OST (represented by thick and thin T bars) [43].

Mammalian cells express two distinct OST complexes that contain STT3A or STT3B as the catalytic subunits and several accessory proteins (Figure 2 and Table 1; STT3A-OST and STT3B-OST) [24,25,26,27]. These accessory proteins include six common subunits (RPN1, RPN2, OST48/DDOST, OST4, TMEM258 and DAD1), STT3A-OST-specific subunits (DC2/OSTC and KCP2) [28] and STT3B-OST-specific subunits (TUSC3 and MAGT1) [17,29]. The two OST complexes are known to have distinct, but partially overlapping specificity to DLO glycans and acceptor sites [23,24,30,31,32,33]. Regarding DLO glycans, it has been reported that in vitro, STT3A-OST shows a strict specificity to the fully assembled DLO, whereas STT3B-OST can also accept DLOs that are completely devoid of glucose residues [24]. The glucose residues of DLO are required for the efficient binding of STT3A-OST, but not STT3B-OST, to acceptor peptides, indicating that the glycan moiety of the fully assembled DLO promotes N-glycosylation by STT3A-OST [24]. STT3 orthologs contain an evolutionarily conserved external loop 5 (EL5), which binds to both donor and acceptor substrates via its N-terminal and C-terminal regions, respectively [34]. It has been proposed that the EL5 loop of *Campylobacter lari* PglB, a bacterial ortholog of STT3, controls the accessibility of the glycan moiety of lipid-linked oligosaccharides to the active site of PglB. Although the precise role of the EL5 loop of mammalian STT3 proteins in catalysis remains unknown, it is attractive to speculate that the microenvironment surrounding the EL5 loop is distinctly different between STT3A-OST and STT3B-OST, which could limit the full activation of STT3A-OST by incompletely assembled DLOs. In support of this hypothesis, DC2, a STT3A-OST-specific subunit, is in contact with the second transmembrane domain of STT3A, which is located close to the EL5 loop [35].

OST catalyzes the transfer of glycans to acceptor sites only when the sites are located in the extended region of a polypeptide [23] and are located a distance of 12–14 amino-acid units from the luminal surface of the ER membrane [36]. Because nascent polypeptides begin the folding process as soon as they emerge into the ER lumen, N-glycosylation needs to occur before the acceptor sites are embedded in the folded region of the proteins. In mammalian cells, STT3A-OST is associated with membrane-bound ribosomes and the protein-conducting channel [23,25,35]. This supra-molecular complex formation enables STT3A-OST to catalyze N-glycosylation coupled with translation and translocation, that is, co-translational N-glycosylation. However, STT3A-OST skips some of the acceptor sites when they are located too close to each other [31], near signal peptides [23], and in the extreme C-terminus of the polypeptide [32]. These skipped sites may be modified by STT3B-OST in a post-translational manner [23], thereby maximizing the efficiency of N-glycosylation. It should be noted that STT3B-OST can also modify some of the STT3A-sites in the absence of STT3A-OST [29], raising the possibility that these sites are located in the polypeptide region that remains as an extended structure. It is also possible that STT3B-OST becomes associated with the translocon to mediate co-translational N-glycosylation in the absence of STT3A-OST. The association of STT3B with the ribosome-associated membrane protein fraction is much weaker than that for STT3A [23], and no clear density of STT3B-OST has been found in the cryo-electron tomography map of the translocon in micorosomes from STT3A-knocked out cells [35]. However, the yeast OST complex, which is classified as a STT3B type [2], has the ability to directly bind to ribosomes in vitro [37]. Given the above findings, we speculate that, in the absence of STT3A-OST, STT3B-OST may be transiently associated with the translocon and mediate co-translational N-glycosylation with a much lower efficiency than STT3A-OST.

Transthyretin (TTR), a soluble secretory protein that is involved in familial amyloidosis, is not modified with N-glycans under normal conditions. Interestingly, however, a disease-related mutation (D18G) of TTR destabilizes the polypeptides and exposes a cryptic N-glycosylation site (N98-D-S), where STT3B-OST adds N-glycans post-translationally. This unusual glycan modification facilitates the clearance of mutant TTR by bringing the protein to the ER-associated degradation (ERAD) machinery [38]. The issue of whether cryptic STT3B-dependent N-glycosylation sites are present in other proteins to promote their ERAD is unclear. Interestingly, however, hemophilia-related missense mutations around the unused N-glycosylation site N582 of coagulation factor VIII (FVIII) have been identified as promoting the N-glycosylation of the site by STT3B, thus impairing the secretion of the aberrantly glycosylated FVIII via, at least in part, the binding of the mutant protein to calreticulin [39,40]. Although the mechanisms by which mutant proteins are trapped in the ER are different between TTR and FVIII, it is reasonable to speculate that STT3B is responsible for the N-glycosylation of cryptic sites that are exposed upon protein-destabilizing, disease-causing mutations. It has also been reported that single-nucleotide variants that occur in somatic cells, for example, tumor cells, can create new N-glycosylation sites in several proteins [41]. It would be interesting to investigate whether these N-glycosylation sites have an impact on tumor progression and which OST complex is responsible for their modification.

A deficiency of either the *STT3A* or *STT3B* gene causes type I congenital disorders of glycosylation (CDGs) with similar symptoms [42], highlighting the need of both N-glycosylation activities for health. The N-glycosylation status of serum transferrin has been used to identify type I CDGs. Transferrin contains two N-glycosylation sites, which are modified by STT3A [32], and is therefore heavily hypoglycosylated in STT3A-CDG [42]. Consistent with this substrate specificity of OST, the N-glycosylation of transferrin is affected only moderately in STT3B-CDG [42]. The identification of other serum glycoproteins that have STT3B-dependent sites will be required for the routine identification of patients with STT3B-CDG.

## 4. Roles of Accessory OST Subunits in N-Glycosylation and Health

Although accessory subunits of OST are required for structural integrity and the maximal activity of OST, their biological roles are not fully understood. Here we summarize key proposed functions of the accessory subunits of OST in N-glycosylation and complex formation (Table 1). Genetic disorders caused by OST deficiency are also discussed.

### 4.1. Shared Subunits

RPN1 and RPN2 (Ost1 and Swp1 in yeast, respectively) are OST subunits shared by both STT3A-OST and STT3B-OST. These proteins were originally identified as potential receptors of membrane-bound ribosomes [44], referred to as “ribophorins”. A cryo-electron microscopy (EM) model of STT3A-OST complexed with the membrane-bound ribosomes and Sec61 protein-conducting channel revealed the presence of a clear contact between the 60S ribosome subunit and the cytosolic domain of RPN1 [35]. In contrast, the density of the cytosolic domain of RPN2 was not visible in the cryo-EM map and the role of this subunit in the binding to ribosomes remains unknown. It should also be noted that RPN1 and RPN2 both contain large luminal domains with no known functions. Interestingly, STT3B-OST contains RPN1, but this OST complex is not associated with the membrane-bound ribosomes [23]. STT3B has a longer cytosolic domain than STT3A, which may interfere with the binding of RPN1 to ribosomes [35].

TMEM258 is a shared subunit of STT3A-OST and STT3B-OST. The *TMEM258* gene was originally identified as a gene associated with Crohn’s disease, rheumatoid arthritis, and elevated arachidonic acid levels in blood [26]. In mice, a haploinsufficiency of *TMEM258* worsens dextran sodium sulfate-induced colitis. TMEM258 is associated with RPN1 and is required for the maximal N-glycosylation of α1 anti-trypsin [26].

DAD1 was initially identified as a negative regulator of apoptosis in temperature-sensitive baby hamster kidney 21 (BHK21) mutant cells [47] and later as an essential OST subunit [48,67]. DAD1 forms heterotetrameric OST subcomplexes with RPN1, RPN2, and OST48/DDOST [67]. Moreover, in BHK21 mutant cells, DAD1 is rapidly degraded at non-permissive temperatures, which decreases the protein stability of RPN1, RPN2, and OST48/DDOST [48]. These findings indicate that DAD1 has a role in maintaining the structural integrity of the OST complex.

OST48/DDOST was first identified as a mammalian ortholog of yeast Wbp1, an essential OST subunit in this organism, and was later found to be associated with the mammalian OST complex [24]. A short cytosolic tail of OST48/DDOST has a functional ER-retention di-lysine motif, serving as a mechanism for retaining OST in the ER [68]. As with the case of DAD1, OST48/DDOST is required for the stability of both STT3A-OST and STT3B-OST [49]. Patients with DDOST-CDG have been identified from untyped CDGs by a combination of whole genome sequencing and biochemical studies [50].

OST4 is a very small, 37 amino acid protein in humans and was identified by its sequence similarity to the yeast ortholog [2]. Although OST4 is associated with both STT3A-OST and STT3B-OST, the knockdown of OST4 destabilizes only STT3A-OST [27]. This is functionally relevant, as the knockdown of OST4 or STT3A, but not STT3B, shows a similar hypoglycosylation phenotype to prosaposin [27], a typical STT3A-dependent substrate [23].

### 4.2. Subunits Specific for STT3A-OST and STT3B-OST

DC2/OSTC and KCP2 are STT3A-OST-specific subunits and are found only in vertebrates [35]. These subunits are not required for the enzymatic activity of STT3A-OST, but are required for the association with the Sec61 channel and, therefore, co-translational N-glycosylation [28,35]. The cryo-EM map of the mammalian ribosome translocon complex located DC2 at the interface between STT3A and Sec61, physically bridging the protein-conducting channel and glycosylation machinery [35]. The mechanism by which STT3B-OST excludes these adaptor proteins is currently unknown, largely due to unavailability of the structural information of STT3B-OST.

STT3B-OST contains either one TUSC3 or one MAGT1 (formerly N33 and IAP [24]) that have a large luminal thioredoxin domain required for N-glycosylation at sites near cysteine residues [33]. Patients with a deficiency in the *MAGT1* and *TUSC3* genes have been reported to show distinct symptoms. Mutations in the *MAGT1* gene are associated with an X-linked immunodeficiency [65] and causes apparent N-glycosylation defects in immunity-related proteins [66]. In contrast, a *TUSC3* deficiency has been associated with autosomal recessive mental retardation with no obvious hypoglycosylation of serum transferrin [51]. Apart from their functions in N-glycosylation, the *TUSC3* and *MAGT1* genes were also identified in a screening of genes that can complement growth defects of yeast mutant cells lacking the plasma membrane Mg^2+^ transporter Alr1 [52]. The *TUSC3* and *MAGT1* genes are reportedly involved in the uptake of Mg^2+^ in mammalian cells, although their Mg^2+^ transport activity has not been demonstrated. The *MAGT1* gene is ubiquitously expressed in various tissues and upregulated when extracellular Mg^2+^ levels are low [52]. On the other hand, the expression of *TUSC3* gene is insensitive to extracellular Mg^2+^ levels and is limited to the ovary, placenta, prostate, testis, adipose tissue, and the lung [52]. The distinct expression patterns of the *MAGT1* and *TUSC3* genes may explain the differences in symptoms of the patients with these genes.

## 5. Roles of OST in Tumor Progression

The N-glycosylation reaction catalyzed by OST has been implicated in the mechanism of immune evasion where tumor cells survive in the tumor microenvironment [9]. The programmed death-ligand 1 (PD-L1) is an immune checkpoint molecule expressed on the surface of various tumor cells and binds to the programmed death-1 (PD-1) receptor on T cells to inactivate them [69]. PD-L1 is a single-pass transmembrane protein with four potential N-glycosylation sites—one (N35) being located at a distance of 17 amino acids from the predicted signal sequence cleavage site, while the other three (N192, N200 and N219) are located adjacent to the transmembrane domain. The addition of glycans to the latter three sites in the ER prevents PD-L1 phosphorylation by glycogen synthase kinase 3β, followed by ERAD, thereby ensuring the cell surface expression of PD-L1 [70].

Epithelial-to-mesenchymal transition (EMT) is a process in which tumor cells lose their contact and acquire migratory and invasive properties by altering protein expression profiles [71,72,73]. Accompanied by the large increase in glycoprotein production in the ER, EMT also programs cells to increase capacity of protein folding and ERAD, and upregulate the metabolic flux of the hexosamine biosynthetic pathway to produce UDP-GlcNAc as substrate for N-glycan biosynthesis [73]. Upon the induction of EMT by transforming growth factor β in breast cancer stem cells, the expression of PD-L1 is upregulated along with STT3A and STT3B through β-catenin signaling [74]. This cell context-dependent regulation of STT3 expression fulfills the N-glycosylation demands of increased PD-L1, thus ensuring the cell surface expression of PD-L1 [74]. As discussed above, however, STT3 proteins require other subunits to be functional, strongly suggesting the presence of mechanisms that control the expression levels of accessory subunits of OST on demand as well. EMT can also influence the expression of glycosyltransferases that are involved in the terminal modification of N-glycans [75], which include the formation of poly-*N*-acetyllactosamine (poly-LacNAc) repeats [76]. Interestingly, β1,3-GlcNAc transferase 3, an enzyme that initiates the formation of poly-LacNAc repeats on N-glycans of PD-L1, is essential for the functional binding of PD-L1 to PD-1 [77]. These findings suggest that EMT reprograms the N-glycosylation machinery of tumor cells toward the activation of PD-L1 to establish PD-L1-mediated immune escape.

## 6. Association of RPN2 and TUSC3 with Tumor Progression

*RPN2* was identified as a gene that confers docetaxel resistance to breast cancers by regulating, as a part of OST, the N-glycosylation of the P-glycoprotein and CD63 [45,46]. The expression of RPN2 is clinically relevant, showing a positive correlation with the degree of aggressiveness of breast cancers in patients [78]. An aberrantly high expression of RPN2 is also associated with the progression of non-small cell lung [79], colorectal [80], gastric [81], and esophageal cancers [82]. RPN2 is also required for the N-glycosylation of the epidermal growth factor receptor (EGFR), aiding the cell surface expression of EGFR and downstream signaling [83]. Although the molecular mechanisms underlying the abnormal expression of RPN2 in various cancer cells are unknown, *RPN2* has been shown to be a target gene of microRNA-128 in colorectal cancer cells [84]. It has been reported that the expression of this small non-coding RNA changes during postnatal development [85,86], raising the possibility that RPN2 expression is cell-context-dependent and regulated dynamically during development and oncogenesis.

TUSC3 has been identified as a candidate tumor suppressor [53]. The *TUSC3* gene is located in a region of chromosome 8p22, which is frequently deleted in various epithelial cancers, including prostate, colon, lung, and liver cancers [54,55]. Moreover, the expression of the *TUSC3* gene can also be downregulated by promoter methylation in the colon, lung, and ovarian cancers [56,57,58]. Suppressing *TUSC3* expression has a large impact on the morphology and homeostasis of the ER [59,60] and promotes EMT and tumor growth [61,62,63]. However, target glycoproteins that are affected by the loss of TUSC3 and are responsible for tumor progression have not been identified. It should be noted that the overexpression of TUSC3 has also been observed in clinical samples of non-small cell lung cancers (NSCLC) compared to benign controls [61,64]. The contradicting expression profiles of *TUSC3* in tumors may reflect the complexity of its gene regulation mechanisms. These collective findings suggest that altered TUSC3 expression has an impact on the glycosylation of a subset of STT3B-dependent substrates, leading to the acquisition of aggressive phenotypes within the established tumors.

## 7. OST as a Potential Druggable Target for Cancer Treatment

Although N-glycosylation represents an attractive target for cancer therapy, drugs that are capable of targeting this pathway have not yet been identified. However, the recent development of a novel class of N-glycosylation inhibitors has provided insights into the clinical relevance of OST in cancer treatment. One inhibitor in this class, termed the N-glycosylation inhibitor 1 (NGI-1), was found in the cell-based high-throughput screening of small compounds that can deactivate an ER-targeted, inactivated form of a luciferase mutant (ERLucT) by inhibiting its N-glycosylation [43]. NGI-1 has the potency to inhibit both OST complexes with a preference for STT3B-OST over STT3A-OST [43,87]. Interestingly, the chemical derivatization of NGI-1 increases the selectivity of the analogs to STT3B-OST [87], raising the possibility of structure-based drug design with the objective of separately modulating the functions of individual OST complexes.

It has been shown that NGI-1 inhibits the proliferation of a subset of non-small cell lung cancer (NSCLC) cells that depend on receptor tyrosine kinases (RTKs) for proliferation, such as EGFR and fibroblast growth factor receptor [87]. The N-glycosylation of human EGFR at Asn420 is known to be a negative regulator of ligand-independent oligomerization and the tyrosine phosphorylation of the receptor [88]. However, blocking N-glycosylation with NGI-1 results in the internalization of EGFR and the disruption of EGFR signaling, ultimately inducing cellular senescence, but not apoptosis [43].

EGFR tyrosine kinase inhibitors (TKIs) have been clinically used to treat NSCLC cells harboring EGFR-activating kinase domain mutations [89]. However, drug resistance develops by acquiring secondary and tertiary mutations in the kinase domain [90,91,92] or the amplification of the hepatocyte growth factor receptor MET [93,94,95], leading to tumor progression. N-glycosylation blockade with NGI-1 is able not only to re-sensitize TKI-resistant NSCLC cells to TKIs, but also to result in the dissociation of EGFR from amplified MET signaling by inducing the internalization of EGFR [96]. Thus, the unique action of NGI-1 may offer therapeutic advantages in the treatment of drug-resistant tumors.

## 8. Concluding Remarks

Genetic and biochemical evidence has demonstrated that N-glycosylation in the ER is essential for homeostasis at the systemic and cellular levels, leading to the assumption that the attachment of N-glycans to proteins takes place constitutively and optimally without strict regulation. However, it has become evident that the expression of OST subunits is cell context-dependent and the regulation involves genetic and epigenetic mechanisms. The on-demand-type regulation of OST expression significantly contributes to tumor progression and possibly to physiological processes where cells need to dramatically change their characteristics.

A novel OST inhibitor has opened up an avenue for fighting incurable drug-resistant tumors. Importantly, the action of NGI-1 is distinct from any other existing anti-cancer drug. However, the efficacy and target molecules in each tumor type remain to be defined if undesired side effects are to be prevented and the effects of OST blockade on possible tumor treatment is to be maximized. In this regard, the toxicity associated with the inhibition of OST should be considered carefully, as OST inhibition affects global N-glycosylation, which may generate undesired proteins, result in detrimental effects to healthy cells, and complicate cancers [97]. Thus, efforts should be made to understand the inhibitory mechanisms of NGI-1, as well as the precise functions of accessory subunits of OST in N-glycosylation and tumor progression, which will accelerate structure-based drug design and discovery.

In summary, OST is now considered to be a gatekeeper of health and tumor progression. Further studies will be necessary to explore molecular links that connect OST to physiology and pathology to expand our knowledge on the functions of this mysterious enzyme.

## Figures and Tables

**Table 1:** Subunit compositions and functions of oligosaccharyltransferase (OST).

OST Subunits	Type of OST	Functions in OST Complexes	Phenotypes Caused by Mutations	Phenotypes Caused by Downregulation	References
STT3A	STT3A-OST	Catalytic subunit, co-translational glycosylation	STT3A-CDG	Impaired co-translational glycosylation	[42]
STT3B	STT3B-OST	Catalytic subunit, post-translational glycosylation	STT3B-CDG	Impaired post-translational glycosylation	[42]
RPN1	Shared	Binding to ribosome	Not known	Reduced expression of STT3A and STT3B	[23,35]
RPN2	Shared	Binding to ribosome?	Not known	Hypoglycosylation of P-glycoprotein and CD63	[44,45,46]
TMEM258	Shared	Association with RPN1	Not known	Increased intestinal inflammation in dextran sodium sulfate-treated haploinsufficient mice	[26]
DAD1	Shared	Stabilization of RPN1, RPN2 and OST48	Increased susceptibility to apoptotic cell death at non-permissive temperature	Reduced expression of STT3A, STT3B, OST48/DDOST and KCP2	[47,48,49]
OST48/DDOST	Shared	Stabilization of STT3A-OST and STT3B-OST	DDOST-CDG	Reduced expression of STT3A, STT3B, DAD1 and KCP2	[49,50]
OST4	Shared	Stabilization of STT3A-OST, but not STT3B-OST	Not known	Hypoglycosylation of prosaposin and reduced expression of STT3A and KCP2	[27]
DC2/OSTC and KCP2	STT3A-OST	Association with the Sec61 protein-conducting channel, co-translational glycosylation	Not known	Impaired co-translational glycosylation	[28,35]
TUSC3	STT3B-OST	Thioredoxin, glycosylation at Cys-proximal sites	Autosomal recessive mental retardation	Impaired Mg^2+^ uptake and tumor progression	[33,51,52,53,54,55,56,57,58,59,60,61,62,63,64]
MAGT1	STT3B-OST	Thioredoxin, glycosylation at Cys-proximal sites	X-linked immunodeficiency	Impaired Mg^2+^ uptake	[33,52,65,66]
